# Clinical Decision Support System for Diabetes Based on Ontology Reasoning and TOPSIS Analysis

**DOI:** 10.1155/2017/4307508

**Published:** 2017-10-26

**Authors:** Rung-Ching Chen, Hui Qin Jiang, Chung-Yi Huang, Cho-Tsan Bau

**Affiliations:** ^1^Department of Information Management, Chaoyang University of Technology, Taichung, Taiwan; ^2^College of Computer and Information Engineering, Xiamen University of Technology, No. 600, Ligong Rd., Jimei District, Xiamen, Fujian, China; ^3^Library, Chienkuo Technology University, Changhua, Taiwan; ^4^Taichung Hospital, Ministry of Health and Welfare, Executive Yuan, Taichung, Taiwan

## Abstract

**Introduction:**

Although a number of researchers have considered the positive potential of Clinical Decision Support System (CDSS), they did not consider that patients' attitude which leads to active treatment strategies or *HbA1c* targets.

**Materials and Methods:**

We adopted the American Diabetes Association (ADA) and the European Association for the Study of Diabetes (EASD) published to propose an HbA1c target and antidiabetic medication recommendation system for patients. Based on the antidiabetic medication profiles, which were presented by the American Association of Clinical Endocrinologists (AACE) and American College of Endocrinology (ACE), we use TOPSIS to calculate the ranking of antidiabetic medications.

**Results:**

The endocrinologist set up ten virtual patients' medical data to evaluate a decision support system. The system indicates that the CDSS performs well and is useful to 87%, and the recommendation system is suitable for outpatients. The evaluation results of the antidiabetic medications show that the system has 85% satisfaction degree which can assist clinicians to manage T2DM while selecting antidiabetic medications.

**Conclusions:**

In addition to aiding doctors' clinical diagnosis, the system not only can serve as a guide for specialty physicians but also can help nonspecialty doctors and young doctors with their drug prescriptions.

## 1. Introduction

The Institute of Medicine [1] defines patient-centered care strategy as “providing care that is respectful of and responsive to individual patient preferences, needs, and values and ensuring that patient values guide all clinical decisions.” Clinical practitioners need to select different drugs to meet the needs of patients. However, patients with type 2 diabetes mellitus exhibit tremendous differences in phenotypes resulting in significant heterogeneity in clinical results. Consequently, clinical practitioners cannot be certain whether a prescription for a particular patient is the best.

Clinical decision support system (CDSS) may help clinicians, patients, and others to suggest patient-appropriate evidence-based treatment options. Ontologies are essential tools for the organization and representation of knowledge [2–7]. Ontologies contain the collection of patients, symptoms, diseases, diagnoses, treatments, and drug information, thereby creating a healing strategy according to patient's requirements to reconfigure a clinical decision support system [8]. Some of the studies suggested using ontologies to build clinical guidelines and care plans [5, 9–12].

In most of the knowledge ontologies, there is a design by the experience of domain experts. For example, Bau et al. [2] used domain ontology and rule reasoning to construct a CDSS for diabetic patients undergoing surgery. They have three main classes in this ontology: disease, management, and patient. The disease class consists of diabetes and comorbidity information. The management class consists of anesthesia, capillary glucose tests, control of DM, medication, no medication, and water restriction information. The patient class consists of the patient clinical profile. The system constructs a clinical decision support system (CDSS) for undergoing surgery based on domain ontology and rules reasoning in the setting of hospitalized diabetic patients. However, the ontology knowledge is built on the experience of clinical practitioners, so it is hard to update these ontologies knowledge when there is a new clinical guideline.

Sherimon and Krishnan [11] had proposed an OntoDiabetic system which an ontology-based clinical decision support system for risk analysis and prediction of diabetes mellitus. The system consists of two main ontologies: the diabetic patient clinical analysis ontology and the semantic profile. The diabetic patient clinical analysis ontology and reasoning rules encapsulate the NICE (National Institute for Health and Care Excellence) guidelines. The OntoDiabetic system calculates the score and predicts the risk of diabetic patients due to smoking, alcohol, physical activity, and sexual and cardiovascular diseases that mainly affect diabetes. Lots of effort was made on OntoDiabetic. What seems to be lacking, however, is that the system cannot provide antidiabetic medications suggestion.

Because there are many types of antidiabetic medications, they need to obtain permission from the government. Although the American Diabetes Association (ADA) and the European Association for the Study of Diabetes (EASD) statements [13, 14] provide 12 types of antidiabetic medications, not every drug can be used.  Table 1 shows eight antidiabetic medications which are commonly used in Japan, Korea, Canada, Italy, and Taiwan from 1998 to 2013 [15–21].

Although a number of researchers have considered the positive potential of CDSS, they did not consider patients' characteristics. For example, at the “patients' attitude” factor, if the patient has highly motivated or excellent self-care capacities, it can use active treatment strategies or *HbA1c* targets. To solve this problem, we proposed a solution in our previous research which adopted the ADA and the EASD standards who published an updated position statement on the management of hyperglycemia in type 2 diabetes to build *HbA1c* target inference module as well as drug knowledge ontology [22]. The system combines fuzzy logic and ontology reasoning to propose an antidiabetic medication recommendation system for patients with T2DM.

In this paper, we will further consider the safety and positivity of *HbA1c* target, and the priority of antidiabetic medication. We use the antidiabetic medication profiles, which are presented by the American Association of Clinical Endocrinologists (AACE) and American College of Endocrinology (ACE) in 2016 [23]. Based on the antidiabetic medication profiles, we used the Technique for Order of Preference by Similarity to Ideal Solution (TOPSIS) to calculate the relative closeness to the ideal solution and thus determined the ranking of antidiabetic medications.

## 2. Materials and Methods

**Figure 1:**
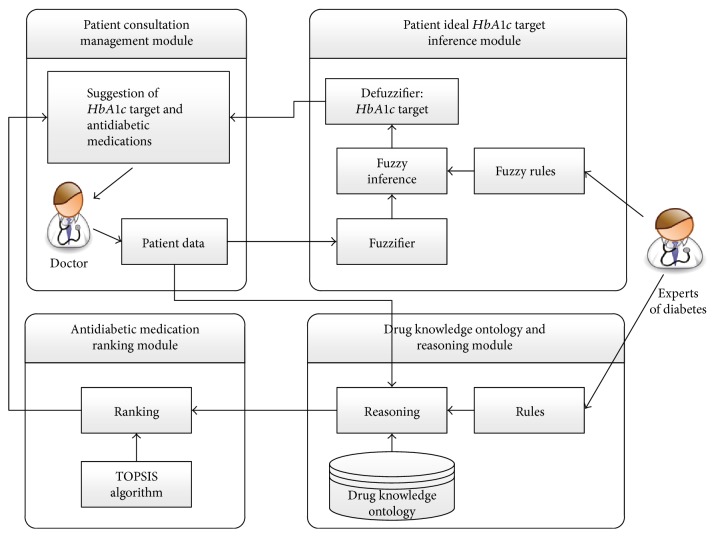
The recommendation system.

The recommendation system consists of four modules: the patient consultation management, the patient perfect *HbA1c* target inference, the drug knowledge ontology and reasoning, and antidiabetic medication ranking modules. The framework of the recommendation system is shown in Figure 1.

The first step, the “patient consultation management module,” provides a user interface to the clinical doctor. So, the clinical doctor can input patient's data in the user interface. Those patient's data will be provided to the other two modules. The second step, the “patient ideal *HbA1c* target inference module,” will use fuzzy technology to infer the patient's individualization HbA1c target. The third step, the “drug knowledge ontology and reasoning module,” will recommend antidiabetic medications for the patient. The fourth step, the “antidiabetic medications ranking module,” will use TOPSIS technology to calculate the relative closeness to the ideal solution and thus determine the ranking of antidiabetic medications. In this system, the experts of diabetes decided the fuzzy rules and ontology reasoning rules.

### 2.1. Patient Consultation Management Module

The patient consultation management module requires the patients' data which is also necessary for the other modules. The modulation of the intensiveness of glucose lowering therapy in T2DM is according to the ADA and the EASD position statement [14]. The sufficient communication between the clinical doctor and the patient is also necessary to evaluate seven factors. They are (1) the risks associated with hypoglycemia and other drug adverse effects, (2) disease duration, (3) life expectancy, (4) important comorbidities, (5) established vascular complications, (6) patient attitude and expected treatment efforts, and (7) resources and support system. Each of the seven factors has five levels measured by integers 0 to 4. The clinical doctor also needs to record adverse drug reactions (ADRs) and individual history of diseases.

### 2.2. Patient Ideal *HbA1c* Target Inference Module

The main functional modules include fuzzifier, fuzzy rules, fuzzy inference, and defuzzier. There are seven inputs, namely, *x*_1_,…, *x*_7_, for fuzzy logic and the input factors are divided into five levels, ranging from integers 0 to 4. The output value *z* is the ideal patient *HbA1c* target level which considers individual differences. The American Association of Clinical Endocrinologists (AACE) and American College of Endocrinology (ACE) suggested *HbA1c* below 6.5% [24], but patient-centered care is needed to consider the patient's characteristics to set the patient's *HbA1c* target. So, the output *z* represents the ideal *HbA1c* target, which varies between 6.5% and 9.0%.

The definition of membership functions is according to the ADA and EASD position statement [14], for example, the “Risks potentially associated with hypoglycemia and other drug adverse effects” can have two levels: “Low” or “High.” So, *x*_1_ has two membership functions: Low(*x*_1_) and High(*x*_1_). The names of the membership functions as well as input and output variables are shown in Table 2. To get the acceptable results, the endocrinologist who works in Taichung Hospital, Ministry of Health and Welfare, has established virtual patients' data and use the FuzzyLite [25] to trial and adjust the parameters of membership functions. Through the sufficient experience of the clinician, the system has better results.

**Figure 2:**
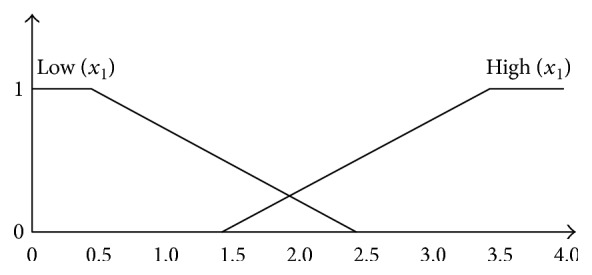
Membership functions of *x*_1_ factor.

**Figure 3:**
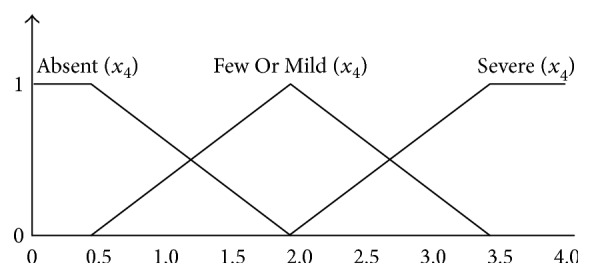
Membership functions of *x*_4_ factor.

**Figure 4:**
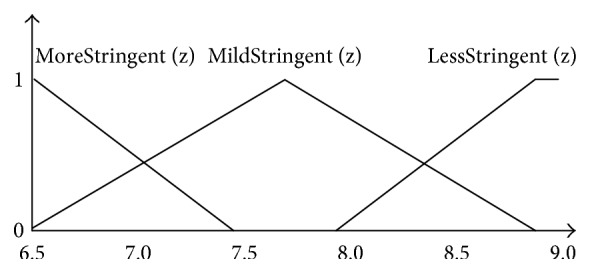
Membership functions of *z* factor.

Figure 2 shows the corresponding membership function for *x*_1_ factor, and the membership function for Low(*x*_1_) and High(*x*_1_) is a trapezoid. Because the *x*_2_, *x*_3_, *x*_6_, and *x*_7_ also can be divided into two functions, their membership functions are the same as that for *x*_1_.  Figure 3 shows the corresponding membership function for *x*_4_ factor, and the membership function for Absent(*x*_4_) and Severe(*x*_4_) is trapezoidal while FewOrMild(*x*_4_) is triangular. Because the *x*_5_ also can be divided into three functions, *x*_5_ membership functions are the same as *x*_4_. The domain knowledge of the defining membership functions is derived from doctors' reports.  Figure 4 shows the corresponding membership function for *z* and the membership functions for MoreStringent(*z*) and LessStringent(*z*) are trapezoidal while that for MildStringent(*z*) is triangular.

The second step is to apply inputs to the fuzzy rules. The fuzzy inference will then stipulate what action will be taken for each combination of sets of memberships. To evaluate the effectiveness of the system, we developed two kinds of fuzzy rule methods. The primary consideration for the first method is relative safety of treatment so we label it “fuzzy safety rules.” The second method is to consider the performance of more positive treatment; we mark it “fuzzy positivity rules.”

#### 2.2.1. Method 1: Fuzzy Safety Rules

The number of fuzzy rules depends on several input factors. For example, if the clinical doctor inputs ×1, ×2, and ×4 values, the fuzzy rules will consist of 12 individual rules. Because ×1 has two membership functions (low and high), ×2 has two membership functions (Newly Diagnosed and Long Standing), and ×4 has three membership functions (Absent, Few or Mild, and Severe). Based on individual experts' experience and intuition, the fuzzy rules table is shown in Table 3. Rule 1 indicates that if *x*_1_ is low and *x*_2_ is newly diagnosed, and *x*_4_ is absent, then *z* is more stringent. Rule 2 states that if *x*_1_ is low and *x*_2_ is newly diagnosed, and *x*_4_ is few or mild, then *z* is mild stringent. Otherwise, the output *z* is less stringent in rules 3–12 because *x*_1_ is high, or *x*_2_ is long standing, or *x*_4_ is severe.

#### 2.2.2. Method 2: Fuzzy Positivity Fuzzy Rules

Table 4 shows the fuzzy positivity rules. Rules 1–5 indicate that if one of the {*x*_1_, *x*_2_, *x*_3_, *x*_4_, *x*_5_} input variables is High/Long-Standing/Short/Severe/Severe, then *HbA1c* is less stringent. Rules 6–9 indicate that if one of the {*x*_2_, *x*_3_, *x*_6_, *x*_7_} input variables is Newly-Diagnosed/Long/Highly-Motivated/Readily-Available, then *HbA1c* is more stringent. Rule 10 indicates that if the “Risks-Of-Hypoglycemia-or-Drug-Effects” are low and “Important-Comorbidities” and “Established-Vascular-Complications” are absent, then *HbA1c* is More-Stringent. Rules 11 and 12 show that if “Important-Comorbidities” or “Established-Vascular-Complications” are Few-or-Mild, then *HbA1c* is Mild-Stringent. Rule 13 states that if both of “Important-Comorbidities” and “Established-Vascular-Complications” are “Few-or-Mild,” then *HbA1c* is Less-Stringent.

Finally, for both fuzzy safety rules and fuzzy positivity rules, the system uses the mean of maximum (MOM) to perform defuzzification.

### 2.3. Drug Knowledge Ontology and Reasoning Module

Protégé and WebProtégé are free software programs for building ontology knowledge solutions [6, 26]. Further, “Jena” is the Java rule-based inference engine developed by Apache Software Foundation [27]. We use WebProtégé to build drug knowledge, and the web-based interface is an easy interface with a diabetes diplomate. When the ontology build is complete, we use Jena to evaluate the antidiabetic medications reasoning module. The details are as follows.

#### 2.3.1. Drug Knowledge Ontology

**Figure 5:**
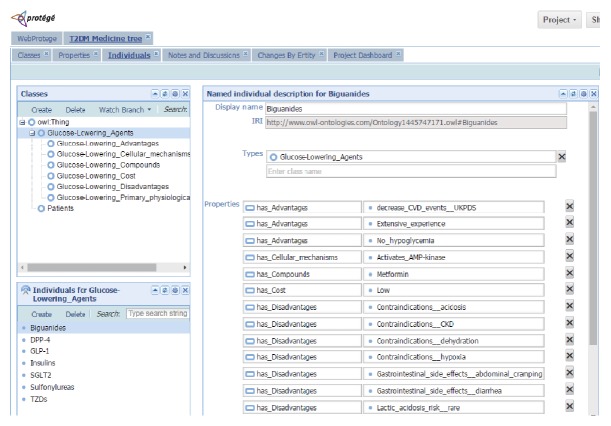
“Biguanides” instances of the “Glucose-Lowering_Agents” class.

**Figure 6:**
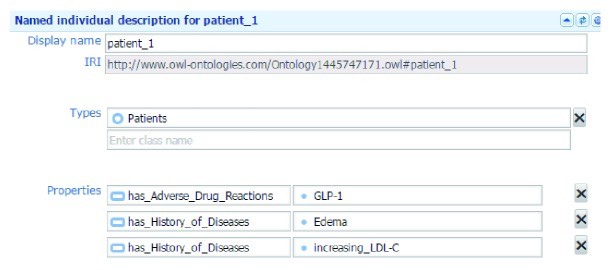
Example of “patient_1.”

According to an update of the position statement published by the ADA and the EASD [13, 14], we created a glucose-lowering agents ontology.  Table 5 shows the classes and the descriptions of their concepts in the domain knowledge. Classes can contain individual objects called “instances.” Table 6 presents the defined properties in the ontology. Object properties represent relationships between two instances and each property has a domain and range. After classes and object properties are created, we build glucose-lowering agent instances based on the ADA and the EASD's position statement on the management of hyperglycemia in type 2 diabetes.  Figure 5 shows “Biguanides” instances of the “Glucose-Lowering_Agents” class and Figure 6 shows an example of patient_1's instance.

#### 2.3.2. Antidiabetic Medications Reasoning Module

Jena is a free and open source Java framework for building semantic web and inference applications [27]. The Jena inference engines support the use of Jena rules to infer from instance data and class descriptions.

Jena is a rule inference engine running on the Java platform. This study developed Jena rules for reasoning which Glucose-Lowering_Agents are not suitable for patients.  Table 7 shows the rules described as follows:

Rule 1: If patients have a history of disease which is related to the disadvantages of Glucose-Lowering_Agents, the Glucose-Lowering_Agents are not recommended.
Rule 2: If patients have adverse drug reactions (ADRs), the ADRs are not recommended.

When the system removes some antidiabetic medications, the system can determine other antidiabetic medications. For example, patient_1 has a history of “increasing_LDL-C” and “Edema.” TZDs has both disadvantages, “increasing_LDL-C” and “Edema,” one of SGLT2's disadvantages is “increasing_LDL-C.” By Rule 1, TZDs and SGLT2 will not be recommended to patient_1. patient_1 also has ADRs with GLP-1. Thus, by Rule 2, GLP-1 will not be recommended to patient_1. This system provides seven common antidiabetic medications in Taiwan which include Biguanides (Metformin), Sulfonylureas (SU), TZDs, DPP-4, SGLT2, GLP-1, and Insulin. When TZDs, SGLT2, and GLP-1 are not recommended to patient_1, Biguanides (Metformin), DPP-4, Sulfonylureas (SU), and Insulin are recommended.

### 2.4. Define Risk of Antidiabetic Medications

The American Association of Clinical Endocrinologists (AACE) and American College of Endocrinology (ACE) published an algorithm for determining glycemic control in 2009 [28]. The comprehensive diabetes management algorithm was updated in 2013, 2015, and 2016 [23, 24, 29–31]. One significant contribution was the presentation of antidiabetic medication profiles. It shows each drug's properties considered for patients.

According to the antidiabetic medication profiles, we convert seven traditional antidiabetic drugs to present the antidiabetic medication risk values.  Table 8 shows the risks of antidiabetic medications. The risk value of “Few adverse events of possible benefits” is 1. The risk value of “Neutral” is 3 and “Use with caution” is 5. Finally, the “Likelihood of adverse effects” is defined as a risk value of 7. Of note, we added “cost” property values according to the position statement of the ADA and the EASD [14]. If cost is “Low,” the property value is 1. If cost is “High,” the property value is 3. In this case, there are some antidiabetic medications like “Meglitinides,” “*α*-glucosidase inhibitors” whose cost is “Moderate” so the property value is 2. However, these antidiabetic medications are not popular in Taiwan, so they do show in Table 8.

### 2.5. TOPSIS Multicriteria Decision Analysis

When the risk of antidiabetic medications is known, we can use it to calculate the antidiabetic medication recommended priority. The Technique for Order of Preference by Similarity to Ideal Solution (TOPSIS) implements a multicriteria decision which was developed by Hwang and Yoon in 1981 [4, 32–34]. TOPSIS was employed to decide antidiabetic medications ranking.

In previous calculations, the system recommended MET (Biguanides), DPP-4, SU (Sulfonylureas), and Insulin for patient_1.  Table 9 shows the risk of antidiabetic medications and cost for patient_1. We will use the risk data of Table 9 as an example to explain the TOPSIS method.

The TOPSIS process of patient_1 was carried out as follows.

**Step 1 : Create the Decision Matrix.**

Create an evaluation matrix consisting of *m* alternatives and *n* criteria with the intersection of each alternative and criteria are given as *A*.

(1) $$\begin{array}{c} A={\left[A_{ij}\right]}_{m\times n}=\left[\begin{array}{ccc} a_{11} & \cdots & a_{1n}\\ \vdots & \ddots & \vdots \\ a_{m1} & \cdots & a_{mn} \end{array}\right],\;i=1,2,\ldots ,m,j=1,2,\ldots ,n. \end{array}$$

For example, the decision matrix of Table 9 is

(2) $$\begin{array}{c} A_{4\times 8}=\left[\begin{array}{cc} \begin{array}{cc} 3 & 1\\ 3 & 3 \end{array} & \begin{array}{cc} 7 & 5\\ 3 & 3 \end{array}\\ \begin{array}{cc} 7 & 7\\ 7 & 7 \end{array} & \begin{array}{cc} 7 & 3\\ 7 & 3 \end{array} \end{array}\;\begin{array}{cc} \begin{array}{cc} 3 & 1\\ 3 & 3 \end{array} & \begin{array}{cc} 3 & 1\\ 3 & 3 \end{array}\\ \begin{array}{cc} 3 & 5\\ 3 & 3 \end{array} & \begin{array}{cc} 3 & 1\\ 3 & 3 \end{array} \end{array}\right]. \end{array}$$

**Step 2 : Construct Normalized Decision Matrix.**

The matrix *A* is then normalized to form the matrix:

(3) $$\begin{array}{c} R={\left[R_{ij}\right]}_{m\times n}=\left[\begin{array}{ccc} r_{11} & \cdots & r_{1n}\\ \vdots & \ddots & \vdots \\ r_{m1} & \cdots & r_{mn} \end{array}\right], \end{array}$$

where $r_{ij}=a_{ij}/\left(\sqrt{\sum_{k=1}^{m}a_{kj}^{2}}\right),i=1,2,\ldots ,m,j=1,2,\ldots ,n$.

(4) $$\begin{array}{c} R_{4\times 8}=\left[\begin{array}{cc} \begin{array}{cc} \begin{array}{cc} 0.279 & 0.096\\ 0.279 & 0.289 \end{array} & \begin{array}{cc} 0.560 & 0.693\\ 0.240 & 0.416 \end{array}\\ \begin{array}{cc} 0.650 & 0.674\\ 0.650 & 0.674 \end{array} & \begin{array}{cc} 0.560 & 0.416\\ 0.560 & 0.416 \end{array} \end{array} & \begin{array}{cc} \begin{array}{cc} 0.500 & 0.151\\ 0.500 & 0.452 \end{array} & \begin{array}{cc} 0.500 & 0.224\\ 0.500 & 0.671 \end{array}\\ \begin{array}{cc} 0.500 & 0.754\\ 0.500 & 0.452 \end{array} & \begin{array}{cc} 0.500 & 0.224\\ 0.500 & 0.671 \end{array} \end{array} \end{array}\right]. \end{array}$$

**Step 3 : Determine the Weight.**

Determine the weight *W* with the antidiabetic medication's risk properties and cost. The initial weight *W* = [*w*_1_ *w*_2_ ⋯ *w*_*n*_] = [1 1 1 1 1 1 1 1]; *w*_1_ is weight of the Hypo property; *w*_2_ is weight of Weight property; *w*_3_ is weight of Renal/GU property; *w*_4_ is weight of GI Sx property; *w*_5_ is weight of CHF property; *w*_6_ is weight of CVD property; *w*_7_ is weight of Bone property; and *w*_8_ is weight of Cost property.

In this case, patient_1 has a history of “Edema” and “increasing LDL-C.” Because “Edema” is relative to CHF, the *w*_5_ is set to 2, and “increasing LDL-C” is relative to CVD, the *w*_6_ set to 2. The weight *W* of patient_1 is shown as follows:

(5) $$\begin{array}{c} W=\left[w_{1}\;w_{2}\;w_{3}\;w_{4}\;w_{5}\;w_{6}\;w_{7}\;w_{8}\right]=\left[1\;1\;1\;1\;2\;2\;1\;1\right]. \end{array}$$

**Step 4 : Construct the Weighted Normalized Decision Matrix.**

Calculate the weighted normalized decision matrix *V*:

(6) $$\begin{array}{c} V={\left[V_{ij}\right]}_{m\times n}=\left[\begin{array}{ccc} v_{11} & \cdots & v_{1n}\\ \vdots & \ddots & \vdots \\ v_{m1} & \cdots & v_{mn} \end{array}\right], \end{array}$$

where *v*_*ij*_ = *w*_*j*_*r*_*ij*_, *i* = 1, 2,…, *m*, and *j* = 1, 2,…, *n*.

(7) $$\begin{array}{c} V_{4\times 8}=\left[\begin{array}{cc} \begin{array}{cc} \begin{array}{cc} 0.279 & 0.096\\ 0.279 & 0.289 \end{array} & \begin{array}{cc} 0.560 & 0.693\\ 0.240 & 0.416 \end{array}\\ \begin{array}{cc} 0.650 & 0.674\\ 0.650 & 0.674 \end{array} & \begin{array}{cc} 0.560 & 0.416\\ 0.560 & 0.416 \end{array} \end{array} & \begin{array}{cc} \begin{array}{cc} 1.000 & 0.302\\ 1.000 & 0.905 \end{array} & \begin{array}{cc} 0.500 & 0.224\\ 0.500 & 0.671 \end{array}\\ \begin{array}{cc} 1.000 & 1.508\\ 1.000 & 0.905 \end{array} & \begin{array}{cc} 0.500 & 0.224\\ 0.500 & 0.671 \end{array} \end{array} \end{array}\right]. \end{array}$$

**Step 5 : Determine the Ideal and Negative Ideal Solutions.**

Determine the ideal solution *A*^∗^ and the negative ideal solution *A*^−^:

(8) $$\begin{array}{c} A^{*}=\left\{V_{1}^{*},V_{2}^{*},\ldots ,V_{n}^{*}\right\}, \end{array}$$

where $V_{j}^{*}=\underset{i}{\min}V_{ij},i=1,2,\ldots ,m$.

(9) $$\begin{array}{c} A^{-}=\left\{V_{1}^{-},V_{2}^{-},\ldots ,V_{n}^{-}\right\}, \end{array}$$

where $V_{j}^{-}=\underset{i}{\max}V_{ij},i=1,2,\ldots ,m.$

(10) $$\begin{array}{c} A^{*}=\left(\underset{i}{\min}v_{i1},\underset{i}{\min}v_{i2},\underset{i}{\min}v_{i3},\underset{i}{\min}v_{i4},\underset{i}{\min}v_{i5},\underset{i}{\min}v_{i6},\underset{i}{\min}v_{i7},\underset{i}{\min}v_{i8}\;\right)=\left(0.279,0.096,0.240,0.416,1.000,0.302,0.500,0.224\right),\\ A^{-}=\left(\underset{i}{\max}v_{i1},\underset{i}{\max}v_{i2},\underset{i}{\max}v_{i3},\underset{i}{\max}v_{i4},\underset{i}{\max}v_{i5},\underset{i}{\max}v_{i6},\underset{i}{\max}v_{i7},\underset{i}{\max}v_{i8}\right)=\left(0.650,0.674,0.560,0.693,1.000,1.508,0.500,0.671\right). \end{array}$$

**Step 6 : Calculate the Separation Measures for Each Alternative.**

Calculate the distance between the target alternative *i* and ideal alternative *S*_*i*_^∗^ and the negative ideal alternative *S*_*i*_^−^:

(11) $$\begin{array}{c} S_{i}^{*}=\sqrt{\overset{n}{\underset{j=1}{\sum }}{\left(V_{ij}-V_{j}^{*}\right)}^{2}},\;i=1,2,\ldots ,m,\\ S_{i}^{-}=\sqrt{\overset{n}{\underset{j=1}{\sum }}{\left(V_{ij}-V_{j}^{-}\right)}^{2}},\;i=1,2,\ldots ,m. \end{array}$$

From the above formula, the system can find the values of *S*_*i*_^∗^ and  *S*_*i*_^−^ as follows:

(12) $$\begin{array}{c} S_{1}^{*}=0.424,\\ S_{2}^{*}=0.775,\\ S_{3}^{*}=1.424,\\ S_{4}^{*}=1.067,\\ S_{1}^{-}=1.458,\\ S_{2}^{-}=0.911,\\ S_{3}^{-}=0.526,\\ S_{4}^{-}=0.664. \end{array}$$

**Step 7 : Calculate the Relative Closeness to the Ideal Solution *C*_*i*_^∗^.**

(13) $$\begin{array}{c} C_{i}^{*}=\frac{S_{i}^{-}}{S_{i}^{*}+S_{i}^{-}},\;i=1,2,\ldots ,m. \end{array}$$

From the above formula, the system will select the options with *C*_*i*_^∗^ closest to 1.

(14) $$\begin{array}{c} C_{1}^{*}=0.775,\\ C_{2}^{*}=0.540,\\ C_{3}^{*}=0.270,\\ C_{4}^{*}=0.384. \end{array}$$

The results show that the ideal solution *C*_1_^∗^ of MET is 0.775, the ideal solution *C*_2_^∗^ of DPP-4 is 0.540, the ideal solution *C*_3_^∗^ of SU is 0.270, and the ideal solution *C*_4_^∗^ of the Insulin is 0.384. Because  *C*_1_^∗^ > *C*_2_^∗^ > *C*_4_^∗^ > *C*_3_^∗^, the recommendation priority of antidiabetic medications is MET > DPP-4 > Insulin > SU.

## 3. Results

According to the ADA and the EASD's statement, one of the major changes in treatment options is a new antidiabetic medication “SGLT2” which is added. So, we add “SGLT2” to this experiment. However, “Meglitinides” and “*α*-Glucosidase” have “Frequent dosing schedule” disadvantage, so we exclude those two antidiabetic medications. Finally, this system provides seven common antidiabetic medication choices which include “Biguanides,” “SU,” “TZDs,” “DPP-4,” “SGLT2,” “GLP-1,” and “Insulin.”

Taichung Hospital is an accredited area hospital in central Taiwan. At the beginning of the system development, the endocrinologist who works in Taichung Hospital set up ten virtual patients' medical data to trial and adjusted clinical decision support system (CDSS). We used Mamdani-type fuzzy inference and mean of maximum (MOM) to perform defuzzification.  Table 10 shows the virtual patient's medical data. In Table 10, the *x*_1_, *x*_2_, *x*_3_,…, *x*_7_, “History of Diseases” and “ADRs” are input variables for diabetes diplomat. The *z* is the fuzzy inference output of the *HbA1c* target which, respectively, are fuzzy safety rules (Method 1) and fuzzy positivity rules (Method 2). The “Recommend antidiabetic medications” shows the recommended medications for patients and the ranking value.

An attending physician, an endocrinologist, and a resident physician evaluated the decision support system for diabetes. They were direct using the system and evaluation it by their clinical experience. They evaluated the system using a 12-question, 5-point survey, regarding satisfaction degree, perceived usefulness, and behavioral intentions (see Table 11). All the scores are expressed as percentage. The evaluation results are shown in Table 12. The clinical decision support system (CDSS) perceived 73% satisfactions. The results of antidiabetic medication recommendation indicate that the system has 70% satisfaction and 71% has intentions to use it.

According to the feedback of resident physicians, because the inpatients may have too many complications, so the resident physicians mostly use insulin to control *HbA1c*. Therefore, the evaluation results of the resident physician will be relatively weak; this is because the recommendation system is only suitable for outpatients. So, if we exclude the results of the assessment of the resident physicians, the evaluation results of the system will be better. In this situation, the participating clinicians have 87% acceptance, and the likelihood of using the system at work and recommending it to others is 77%.

The fuzzy safety rules (Method 1) has 80% accuracy and satisfaction, but the fuzzy positivity rules (Method 2) is only 60%. So, the fuzzy safety rules (Method 1) is better than the fuzzy positivity rules (Method 2) for the patient ideal HbA1c target inference. The evaluation result of “Antidiabetic medications reasoning and ranking” shows that the system has 85% satisfaction which can assist clinicians to the management of T2DM while selecting antidiabetic medications.

**Figure 7:**
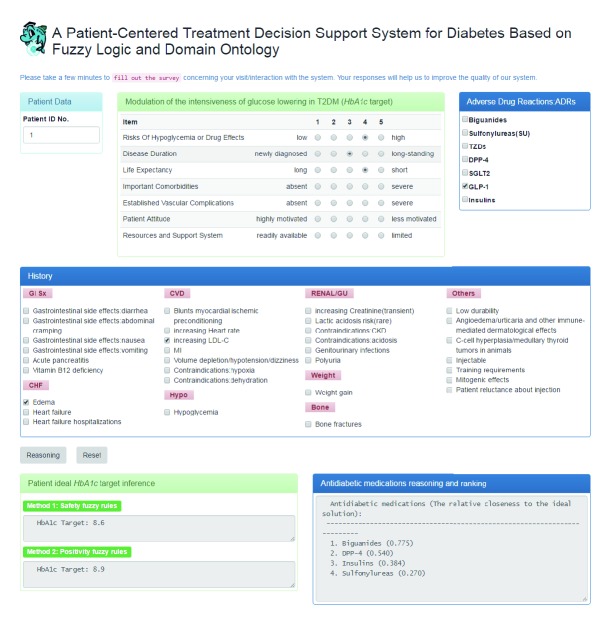
User interface for the antidiabetic medication recommendation system.

The user interface for the antidiabetic medication recommendation system is shown in Figure 7. The website of the system is http://120.109.46.42/T2DM/. Doctors may refer to the system to make prescriptions. Of course, the system, which is likely to make misleading or inappropriate suggestions, cannot replace a doctor's clinical experience and professional judgment. The doctor thus makes the final decision.

## 4. Discussion

The number of patients with diabetes worldwide is significant and continually increasing. Diabetes imposes psychological, physical, and financial hardship on patients. Diabetes therapy, no doubt, is a complicated task. As regards the prescription strategy of clinical doctors, it is necessary that they consider many factors. However, the following two reasons will affect the doctor's decision to use the system:

1. If a doctor uses the new and expensive drugs as a treatment prescription, he will worry that the health insurance will not pay medical expenses. Then, he will only use the generic antidiabetic drugs.
2. The system only provides a single drug treatment prescription, for long-term diabetes patients may need a multidrug treatment prescription to reduce HbA1c effectively.

Even so, CDSS is used to assist humans in making decisions rather than replacing human decisions. The system shows the following clinical values:

- Define appropriate therapeutic goals implementing patient-centered medical care and prescriptions:
- The patient-centered management strategy, by contrast, holds that not all patients can benefit from active glucose management. It stresses individualized therapeutic goals. However, diabetes, multiple complications, and the complexity inherent in antidiabetic medication use often make it difficult for doctors, especially young doctors, to select the best therapeutic strategy. Despite their awareness of the concept of “patient-centered management strategy,” it has shown the difficulty in practice. Given this, we systematized the constructs to help doctors develop their therapeutic goals and selection of prescriptions to meet the patient's needs. In addition to encouraging patients to follow doctors' instructions, this method can also reduce the risks resulting from medical treatment. Therapeutic goals may thus be achieved.
- Doctors can save time and make the best use of medical resources:
- The increasing number of patients with type 2 diabetes has been exhausting medical resources. This system can enable doctors to spend less time on medical diagnosis and adjustment of patients' prescriptions. This will reduce the impact on health care resources.
- Doctors can employ the system with ease, and their clinical inertia can be reduced:
- This system is manipulation-friendly. By inputting a few needed parameters, doctors can obtain recommended antidiabetic medications in order of effectiveness and thus make their treatment judgment accordingly. The system enables physicians to save time in answering patients' questions and can reduce the risk of developing clinical inertia.

## 5. Conclusion and Future Work

The prescription strategy of clinical doctors must take many factors into account. To address this, we developed an individualized antidiabetic medication recommendation system for patients with diabetes. This system, which can be manipulated with relative ease, tailors *HbA1c* levels to mitigate patients' differences. Currently, 12 kinds of antidiabetic medications, both oral and injected, are available. Manually considering all possible conditions is not only a waste of medical resources but also a burden on the system, not to mention that it is impractical. This study, which combines fuzzy logic and ontology reasoning, proposes an antidiabetic medication recommendation system for patients with diabetes. It promotes a new concept of “patient-centered diabetes therapy.” Antidiabetic medications are recommended for the outpatients, and useful ranking of medications is conducted. In addition to aiding doctors' clinical diagnosis, the system can not only serve as a guide for doctors specializing in diabetes but also help family practitioners and interns in prescribing medications.

Based on results of the study using the feedback system of operations, the seven factors analyzed can provide dynamic correlations. We will improve our system interface and dynamic weighting calculations in future research. Besides, we propose an architecture based on rules to build an antidiabetic medication recommendation system. In the future, we will combine rule-based and case-based reasoning to solve the special case issues.

## Figures and Tables

**Table 1:** Utilization of antidiabetic medications from 1998 to 2013.

Authors	(Publication year) title	Source period (year)	Country	Antidiabetic medications
Chang et al. [15]	(2012) National trends in antidiabetic treatment in Taiwan, 2000–2009	2000–2009	Taiwan	Biguanides, SU, Meglitinides, TZDs, *α*-glucosidase, DPP-4, insulin
Abdelmoneim et al. [20]	(2013) Use patterns of antidiabetic regimens by patients with type 2 diabetes	1998–2010	Canada	Biguanides, SU, Meglitinides, TZDs, *α*-glucosidase, insulin
Kohro et al. [17]	(2013) Trends in antidiabetic prescription patterns in Japan from 2005 to 2011—impact of the introduction of dipeptidyl peptidase-4 inhibitors	2005–2011	Japan	Biguanides, SU, Meglitinides, TZDs, *α*-glucosidase, DPP-4, GLP-1, insulin
Hsu et al. [21]	(2015) Utilization of oral antidiabetic medications in Taiwan following strategies to promote access to medicines for chronic diseases in community pharmacies	2001–2010	Taiwan	Biguanides, SU, Meglitinides, TZDs, *α*-glucosidase, DPP-4, GLP-1
Rafaniello et al. [18]	(2015) Trends in the prescription of antidiabetic medications from 2009 to 2012 in a general practice of Southern Italy: a population-based study	2009–2012	Italy	Biguanides, SU, Meglitinides, TZDs, *α*-glucosidase, DPP-4, GLP-1, insulin
Ko et al. [19]	(2016) Trends of antidiabetic drug use in adult Type 2 diabetes in Korea in 2002–2013: nationwide population-based cohort study	2002–2013	Korea	Biguanides, SU, Meglitinides, TZDs, *α*-glucosidase, DPP-4, insulin
Ou et al. [16]	(2016) Recent trends in the use of antidiabetic medications from 2008 to 2013: a nationwide population-based study from Taiwan	2008–2013	Taiwan	Biguanides, SU, Meglitinides, TZDs, *α*-glucosidase, DPP-4, GLP-1, insulin

SU: sulfonylureas; TZDs: thiazolidinediones; DPP-4: dipeptidyl peptidase-4; SGLT2: sodium-glucose cotransporter 2; GLP-1: glucagon-like peptide 1.

**Table 2:** Names of membership functions, input, and output variables.

Variable	Name	Function 1	Function 2	Function 3
*x*_1_	Risks potentially associated with hypoglycemia and other drug adverse effects	Low	High	—
*x*_2_	Disease duration	Newly Diagnosed	Long Standing	—
*x*_3_	Life expectancy	Long	Short	—
*x*_4_	Important comorbidities	Absent	FewOrMild	Severe
*x*_5_	Established vascular complications	Absent	FewOrMild	Severe
*x*_6_	Patient attitude and expected treatment efforts	Highly Motivated	Less Motivated	—
*x*_7_	Resources and support system	Readily Available	Limited	—
*z*	*HbA1c*	More Stringent	Mild Stringent	Less Stringent

**Table 3:** Example of fuzzy safety rules table.

Rule	*x*_1_	*x*_2_	*x*_4_	*z*
1	Low	Newly Diagnosed	Absent	More Stringent
2	Low	Newly Diagnosed	FewOrMild	Mild Stringent
3	Low	Newly Diagnosed	Severe	Less Stringent
4	Low	Long Standing	Absent	Less Stringent
5	Low	Long Standing	FewOrMild	Less Stringent
6	Low	Long Standing	Severe	Less Stringent
7	High	Newly Diagnosed	Absent	Less Stringent
8	High	Newly Diagnosed	FewOrMild	Less Stringent
9	High	Newly Diagnosed	Severe	Less Stringent
10	High	Long Standing	Absent	Less Stringent
11	High	Long Standing	FewOrMild	Less Stringent
12	High	Long Standing	Severe	Less Stringent

**Table 4:** Example of fuzzy positivity rules table.

Rule	Function
1	If (Risks-Of-Hypoglycemia-or-Drug-Effects is High) then (*HbA1c* is Less-Stringent)
2	If (Disease-Duration is Long-Standing) then (*HbA1c* is Less-Stringent)
3	If (Life-Expectancy is Short) then (*HbA1c* is Less-Stringent)
4	If (Important-Comorbidities is Severe) then (*HbA1c* is Less-Stringent)
5	If (Established-Vascular-Complications is Severe) then (*HbA1c* is Less-Stringent)
6	If (Disease-Duration is Newly-Diagnosed) then (*HbA1c* is More-Stringent)
7	If (Life-Expectancy is Long) then (*HbA1c* is More-Stringent)
8	If (Patient-Attitude is Highly-Motivated) then (*HbA1c* is More-Stringent)
9	If (Resources-and-Support-System is Readily-Available) then (*HbA1c* is More-Stringent)
10	If (Risks-Of-Hypoglycemia-or-Drug-Effects is Low) and (Important-Comorbidities is Absent) and (Established-Vascular-Complications is Absent) then (*HbA1c* is More-Stringent)
11	If (Important-Comorbidities is Few-or-Mild) then (*HbA1c* is Mild-Stringent)
12	If (Established-Vascular-Complications is Few-or-Mild) then (*HbA1c* is Mild-Stringent)
13	If (Important-Comorbidities is Few-or-Mild and (Established-Vascular-Complications is Few-or-Mild) then (*HbA1c* is Less-Stringent)

**Table 5:** Classes in the domain ontology.

Class	Description
Glucose-Lowering_Agents	Concepts are glucose-lowering drugs. Ontology content is based on the ADA/EASD's position statement on management of hyperglycemia in type 2 diabetes to be established
Glucose-Lowering_Advantages	Concepts about glucose-lowering advantages
Glucose-Lowering_Cellular_mechanisms	Concepts about glucose-lowering cellular mechanisms
Glucose-Lowering_Compounds	Concepts about glucose-lowering compounds
Glucose-Lowering_Cost	Concepts about glucose-lowering cost
Glucose-Lowering_Disadvantages	Concepts about glucose-lowering disadvantages
Glucose-Lowering_Primary_physiological_actions	Concepts about glucose-lowering primary physiological actions
Patients	Concepts about patient's profile, the properties include patient's adverse drug reactions (ADRs) and history of diseases

**Table 6:** Defined properties in the ontology.

Property name	Property type	Domain	Range
has_Advantages	Object	Glucose-Lowering_Agents	Glucose-Lowering_Advantages
has_Cellular_mechanisms	Object	Glucose-Lowering_Agents	Glucose-Lowering_Cellular mechanisms
has_Compounds	Object	Glucose-Lowering_Agents	Glucose-Lowering_Compounds
has_Cost	Object	Glucose-Lowering_Agents	Glucose-Lowering_Cost
has_Disadvantages	Object	Glucose-Lowering_Agents	Glucose-Lowering_Disadvantages
has_Primary_physiological_actions	Object	Glucose-Lowering_Agents	Glucose-Lowering_Primary physiological_actions
has_History_of_Diseases	Object	Patients	Glucose-Lowering_Disadvantages
has_Adverse Drug_Reactions	Object	Patients	Glucose-Lowering_Agents
Not_recommended	Object	Patients	Glucose-Lowering_Agents
ID_No	Data	Patients	xsd: string

**Table 7:** Example of ontology reasoning rules table.

No.	Rule
(1)	(?x rdf:type http://www.owl-ontologies.com/Ontology1445747171.owl#Patients) (?x http://www.owl-ontologies.com/Ontology1445747171.owl#has_History_of_Diseases ?y) (?n rdf:type http://www.owl-ontologies.com/Ontology1445747171.owl#Glucose-Lowering_Agents) (?n http://www.owl-ontologies.com/Ontology1445747171.owl#has_Disadvantages ?y) -> (?x http://www.owl-ontologies.com/Ontology1445747171.owl#Not_recommand ?n)
(2)	(?x rdf:type http://www.owl-ontologies.com/Ontology1445747171.owl#Patients) (?x http://www.owl-ontologies.com/Ontology1445747171.owl#has_Adverse_Drug_Reactions ?n) -> (?x http://www.owl-ontologies.com/Ontology1445747171.owl#Not_recommand ?n)

**Table 8:** Risk of antidiabetic medications and cost.

Properties	Antidiabetic medications						
	MET	GLP-1	SGLT2	DPP-4	TZD	SU	Insulin
Hypo	3	3	3	3	3	7	7
Weight	1	1	1	3	5	7	7
Renal/GU	7	7	5	3	3	7	7
GI Sx	5	5	3	3	3	3	3
CHF	3	3	3	3	5	3	3
CVD	1	3	3	3	3	5	3
Bone	3	3	3	3	5	3	3
Cost	1	3	3	3	1	1	3

MET: metformin (Biguanides); SU: sulfonylureas; Hypo: hypoglycemia; GU: genitourinary; GI Sx: glycemic index symptom; CHF: congestive heart failure; CVD: cardiovascular diseases.

**Table 9:** Risk of antidiabetic medications and cost for patient_1.

Antidiabetic medications	Properties							
	Hypo	Weight	Renal/GU	GI Sx	CHF	CVD	Bone	Cost
MET	3	1	7	5	3	1	3	1
DPP-4	3	3	3	3	3	3	3	3
SU	7	7	7	3	3	5	3	1
Insulin	7	7	7	3	3	3	3	3

**Table 10:** Ten virtual patients' medical data.

ID	Age	Sex	*x*_1_, *x*_2_, *x*_3_, *x*_4_, *x*_5_, *x*_6_, *x*_7_	has_History of Diseases	has_ADRs	*z* (method 1)	*z* (method 2)	Recommended antidiabetic medications
1	73	Female	3, 2, 3, NaN, NaN, NaN, NaN	increasing_LDL-C, Edema	GLP-1	8.6	8.9	(1) Biguanides (0.775) (2) DPP-4 (0.540) (3) Insulins (0.384) (4) Sulfonylureas (0.270)
2	75	Female	3, 2, 4, NaN, NaN, NaN, NaN	Heart_failure, increasing_LDL-C	NaN	8.6	9.0	(1) Biguanides (0.788) (2) DPP-4 (0.549) (3) GLP-1 (0.536) (4) Insulins (0.376) (5) Sulfonylureas (0.248)
3	64	Female	2, 1, 2, NaN, NaN, NaN, NaN	Bone_fractures, increasing_LDL-C	NaN	6.9	6.6	(1) Biguanides (0.788) (2) DPP-4 (0.549) (3) GLP-1 (0.536) (4) Insulins (0.376) (5) Sulfonylureas (0.248)
4	76	Female	4, 3, 3, 2, 1, NaN, NaN	increasing_LDL-C, Contraindications_CKD	DPP-4	8.8	7.8	(1) GLP-1 (0.631) (2) Insulins (0.445) (3) Sulfonylureas (0.369)
5	61	Female	4, 3, 2, 3, 2, NaN, NaN	Heart_failure, increasing_LDL-C, Contraindications_CKD, Weight_gain	NaN	8.6	7.8	(1) GLP-1 (0.534) (2) DPP-4 (0.466)
6	64	Female	2, 1, 1, NaN, NaN, 2, NaN	NaN	NaN	6.9	6.6	(1) Biguanides (0.731) (2) SGLT2 (0.648) (3) DPP-4 (0.619) (4) GLP-1 (0.586) (5) TZDs (0.549) (6) Sulfonylureas (0.377) (7) Insulins (0.365)
7	62	Male	2, 2, 3, NaN, NaN, 3, 1	Gastrointestinal_side_effects_abdominal_cramping, increasing_LDL-C	NaN	8.6	6.6	(1) DPP-4 (0.703) (2) GLP-1 (0.543) (3) Insulins (0.481) (4) Sulfonylureas (0.417)
8	81	Female	4, 3, 4, 4, 4, 4, 2	MI, increasing_LDL-C, Contraindications_CKD	DPP-4	8.6	9.0	(1) GLP-1 (0.631) (2) Insulins (0.445) (3) Sulfonylureas (0.369)
9	48	Female	1, 1, 2, 3, NaN, NaN, 1	Patient_reluctance_about_injection, increasing_LDL-C	NaN	7.9	6.6	(1) Biguanides (0.796) (2) GLP-1 (0.560) (3) DPP-4 (0.558) (4) Sulfonylureas (0.248)
10	56	Male	NaN, 2, 2, 2, 1, 1, NaN	Weight_gain, increasing_LDL-C, Gastrointestinal_side_effects_nausea	TZDs	7.9	7.8	(1) Biguanides (0.687) (2) DPP-4 (0.313)

**Table 11:** Survey of “Patient-Centered Treatment Decision Support System for Diabetes Based on Fuzzy Logic and Domain Ontology”.

Question	Scoring				
	1	2	3	4	5
What do you think about “Patient ideal *HbA1c* target inference”? Are you satisfied with its accuracy?					
Is the “Method 1: Safety fuzzy rules” accurate?	□	□	□	□	□
Are you satisfied with the results of the “Method 1: Safety fuzzy rules”?	□	□	□	□	□
Is the “Method 2: Positivity fuzzy rules” accurate?	□	□	□	□	□
Are you satisfied with the results of the “Method 2: Positivity fuzzy rules”?	□	□	□	□	□
What do you think about “Antidiabetic medications reasoning and ranking”? Are you satisfied with its accuracy?					
Is the “Antidiabetic medications reasoning and ranking” accurate?	□	□	□	□	□
Are you satisfied with the results of the “Antidiabetic medications reasoning and ranking”?	□	□	□	□	□
Do you think the system can provide some benefits for you?					
Using the system improves my performance in my job.	□	□	□	□	□
Using the system enhances my effectiveness in my job.	□	□	□	□	□
I find the system to be useful in my job.	□	□	□	□	□
If this system used in conjunction with the actual work, would you continue to use this system at work?					
I enjoy using this system at work.	□	□	□	□	□
I will frequently use this system in the future.	□	□	□	□	□
I will strongly recommend to others to use this system.	□	□	□	□	□

Title: ○Endocrinologists ○Attending physicians ○Resident physicians

Gender: ○Male ○Female

E-mail:

**Table 12:** The evaluation result of the system.

Scores	Participants	
	Endocrinologist Attending physician Resident physician	Endocrinologist Attending physician
“*HbA1c* target inference (Safety fuzzy rules: Method 1)” Satisfaction degree (%)	67%	80%
“*HbA1c* target inference (Positivity fuzzy rules: Method 2)” Satisfaction degree (%)	67%	60%
“Antidiabetic medications reasoning and ranking” Satisfaction degree (%)	70%	85%
Perceived usefulness (%)	73%	87%
Intentions to use (%)	71%	77%
